# The effect of the staining technique on morphological and morphometric parameters of boar sperm

**DOI:** 10.1371/journal.pone.0214243

**Published:** 2019-03-25

**Authors:** Magdalena Czubaszek, Katarzyna Andraszek, Dorota Banaszewska, Renata Walczak-Jędrzejowska

**Affiliations:** 1 Department of Animal Genetics and Horse Breeding, Siedlce University of Natural Sciences and Humanities, Siedlce, Poland; 2 Department of Breeding Methods and Poultry Breeding, Siedlce University of Natural Sciences and Humanities, Siedlce, Poland; 3 Department of Andrology and Repoductive Endocrinology, Medical University of Łodz, Łodz, Poland; University Hospital of Münster, GERMANY

## Abstract

Sperm morphology and morphometry are important parameters in predicting fertility. Sperm are considered to be normal if the shape and size of the head, midpiece and tail fall within the classification for a given species. It is important to select the appropriate technique for staining the semen of a given species, because, as many authors have pointed out, some methods work well for one species but are not suitable for analysing another. The aim of the study was to assess the morphometric parameters of boar sperm following the use of different staining techniques and to verify the hypothesis that the staining technique affects the morphometric parameters of sperm. The staining method was found to significantly affect the dimensions of the boar sperm head. The semen stained by the SpermBlue technique had the closest morphometric sperm head parameters to those of the unstained sperm, so this technique, rather than the routinely used eosin and gentian complex, should be the leading technique in the evaluation of boar sperm morphometry. Silver nitrate staining reveals the structure of the sperm in the most detail; this method can be considered universal, and can be used independently or to supplement routine diagnostics. As the staining technique should interfere as little as possible with the structure of the sperm, while revealing its morphology in as much detail as possible, it is crucial to establish the natural dimensions of the unstained sperm head before determining the optimal technique and its reference values. The recommended or most commonly-used techniques are not always the best options for the staining and analysis of sperm of a given species.

## Introduction

A precise diagnosis of ejaculates is necessary to predict male fertility, in both humans and animals, and is important in optimizing and maximizing their reproductive ability for natural conception as well as in assisted reproduction techniques (ART).[1] While other basic semen parameters i.e. motility and total sperm count, are important in predicting fertility, the morphological structure of spermatozoa seems to be the most significant factor, especially for natural conception and artificial insemination.[2–6] It has been shown that spermatozoa with abnormal morphology are not able to reach the oocyte.[7] Also spermatozoa with normal motility but with head defects are incapable of fertilization.[8]

Microscopic analysis of ejaculates shows that sperm morphology is not uniform, even within the same ejaculate, and this creates difficulties during fertility diagnostics.[9,10] Therefore, it is necessary to develop methods of sperm morphology assessment suitable for different species and to standardize them.[11] Ideally, a complete morphological analysis of the male gamete should clearly indicate its fertilization capacity.[12]

In all species, spermatozoa are considered normal if they fall within the classification for a given species, including the shape and size of the head, midpiece and tail.[13] Such a standard exists for human sperm (WHO 2010) [14], but none has been developed for any animal species. The evaluation of sperm morphology is closely related to sperm morphometry, which has been directly linked to the fertility and rabotential of males.[15–18] Clinical studies have shown that the spermatozoa of infertile men have larger heads and a higher ratio of sperm head length to width.[19] Similarly, differences in the dimensions of spermatozoa heads among fertile and infertile males or those with reduced fertility have been found in bulls, stallions, pigs and dogs.[20–23]. There are two theories regarding the shape of the sperm head. Thurston et al. [24] maintain that it is genetically determined, primarily during spermatogenesis; however, this view is increasingly being questioned. The shape of the head may depend primarily on epigenetic factors and is determined during spermatogenesis. Morphologically varied gametes have been shown to appear at this stage, when the genetic factor significantly affects the structure and size of the cell. In addition, the genetic factor is modified by environmental factors and by the process of histone replacement with protamines; the authors argue that an abnormal head shape is associated with impaired chromatin condensation [25]. Sperm with an abnormal head shape may consequently have functional disorders such as a dysfunctional chromatin structure or DNA fragmentation.[26–31]

Therefore, the utilization of morphometric reference values of spermatozoa may increase knowledge of its capacity for natural and *in vitro* fertilization, as well as its quality and function after cryopreservation.[22,32] Moreover, morphological and morphometric evaluation of spermatozoa acrosome structure enables more accurate prediction of fertilization capacity in humans.[33–35] In this context, determination of the morphological structure of the spermatozoa head is of particular importance, because its size and shape are important criteria in the classification of spermatozoa as normal or abnormal ones. However, not only the size of the head affects fertilization capacity, but also the dimensions and function of the tail and midpiece; for example, drone sperm cells with a longer tail have greater fertilization potential due to their increased motility.[36]

The search for the best method for evaluating sperm morphology has led to the use of numerous staining techniques. None of these techniques, however, are error-free. Differences in the results of the sperm morphology assessment using different staining methods can reach even 30–60%.[37–39] It is well documented that the accuracy of sperm morphology evaluation depends on the care taken in slide preparation, fixation and the choice of staining method.[40–45] Although some studies suggest that alternative staining techniques produce comparable results, others have shown significant differences in the intensity of staining and contrast, and most importantly, in the size and shape of the spermatozoa [42–47], and each parameter evaluated can have a significant effect on the morphological assessment.[48] These minor differences in staining techniques are particularly problematic in the evaluation of fertility disorders in cases where the morphological parameters fluctuate within reference values.[48] This increases the importance of the choice of staining technique. Ideally, the method used should interfere as little as possible with the structure and size of the spermatozoon, while also clearly showing the boundaries of its head, midpiece and tail.[12]

In routine evaluation of the morphology of cattle and pig spermatozoa, eosin + gentian complex and eosin + nigrosin complex are the most commonly used stains. According to Kondracki et al. [49,50] and Banaszewska et al. [10], this type of staining is a standard technique for assessing the semen morphology of males used for insemination. In addition, the technique with eosin+ nigrosin can also be used to identify live and dead spermatozoa.[51–53] Staining with eosin + gentian complex accurately reveals the outline of the sperm head, but causes difficulties in observing the extent of the acrosome and the midpiece in stallions and bulls.[42–45] For human semen samples, the modified Papanicolaou staining method is believed to give the best staining pattern and no background staining, and is recommended by the WHO for sperm morphology assessment; however, this technique is very time consuming, as it requires the use of many chemicals including five dyes in three dilutions and more than 20 processing steps. In addition, the method does not produce the desired results in the case of bull or stallion semen.[42,44]

Another staining method used for evaluation of human sperm morphology is Rapidiff, a fast and simple technique (known also as Diff-Quik). The procedure was introduced by Kruger et al. [54], and has been found to be comparable with the Papanicolaou staining technique. Unfortunately, it causes background staining and sperm head swelling.[55]

A simple and fast staining technique for human and animal sperm morphology is SpermBlue. It is recommended for both fresh and frozen semen, and some researchers suggest that this technique produces better results than Papanicolaou or other staining techniques.[56] In animal sperm, this method stains sperm heads very well, but there are problems with analysing the midpiece and tail, because these areas stain less intensely. Similarly to Papanicolau, this method does not stain the background, which could mask certain boundaries in the sperm cell and thereby impede their analysis.[42,44]

In the present study, an experimental technique of staining spermatozoa with silver nitrate colloid solution was used. As silver nitrate is an alkaline dye, it is mainly used to identify acidic chromatin proteins and the chromatin of nucleolus organizer regions in mitotic chromosomes [57–60] and nucleoli during meiosis [61,62] The basic methodology has been modified in our Department (Department of Animal Genetics and Horse Breeding, University of Natural Sciences and Humanities in Siedlce) and successfully used to identify morphological details of the spermatozoa either from fresh, frozen or fixed semen sample of mammals [42–44,63,64], birds [47] and insects.[36]

This staining clearly shows the boundary between the acrosomal region, which stains lighter, and the post-acrosomal region, which stains darker. The latter contains residues of acidic proteins and nucleoli that positively react with silver salts.[63] In addition, the procedure is simple, short and inexpensive.

Unfortunately, unlike human semen evaluation, where WHO recommendations are standard worldwide, there are no such clear recommendations for the assessment of semen samples of different animal species. Thus the evaluation of animal sperm morphology and morphometry is faced by a lack of standardization. According to the Society for Theriogenology (SFT), analysis of stallion sperm morphology should be performed on fresh, unstained specimens using a light microscope with phase contrast.[65] Unfortunately, most laboratories do not have a high-grade phase contrast microscope, and the stallion semen is evaluated mainly after staining with eosin and gentian complex, as recommended by the SFT for the evaluation of bull sperm.[66] The accuracy of the sperm morphology evaluation depends on the care taken in slide preparation, fixation and staining, because this affects the morphometry of the head and the entire sperm.[32–35,40–42] This increases the importance of the choice of staining technique; the method used should interfere as little as possible with the structure of the cells, while at the same time clearly showing the boundaries of the head and other elements of the sperm structure so that each of these parts can be accurately identified.[4]

Researchers increasingly stress the sensitivity and suitability of sperm morphological assessment as a prognostic factor in diagnosing fertility, especially when using the strict Tygerberg criteria for characterizing the sperm head.[43–45,56,67,68] The Tygerberg criteria specify four shape indices of the spermatozoa head: ellipticity, elongation, roughness and regularity. The ellipticity index differentiates thin and conical sperm heads, with higher index values indicating a thinner sperm head. Elongation defines the degree of rounding of the head. If the value is zero, the heads are round. The roughness index identifies heads with an uneven cell membrane surface, sometimes referred to as amorphous, with a lower value indicating a rougher surface to the head. Regularity defines the correctness of the sperm head shape and identifies pear-shaped heads.[4,48]. It has been demonstrated that an abnormal sperm head shape, associated, for example, with disturbed chromatin condensation, may result in the presence in the semen of spermatozoa with elongated and narrowed heads. In this context, assessment of sperm structure is of particular importance, because the size and shape of the head are important criteria in the classification of morphologically-correct sperm or for identifying irregularities in their morphology in order to determine their fertilizing capacity.[4]

Sperm morphology can be evaluated using a number of chemical, biochemical and microscopic techniques. The main problem is that the use of different methods for a given material or type of analysis causes discrepancies in the number of morphologically normal or abnormal sperm identified and in their dimensions and morphometric indices. As a consequence, a male examined in one laboratory can be classified as having normal sperm morphology, while in another it may be identified as an individual with fertility disorders.[10] This is a major obstacle for doctors of human and animal medicine comparing the results of semen analysis from laboratories using different techniques.

Hence, as mentioned above, the key problem faced when evaluating of sperm morphology and morphometry is the lack of standardization with respect to the choice of staining techniques. The use of dyes with different pH, osmolarity and procedure length may affect the shape and size of spermatozoa, and thus the result of the sperm morphology evaluation. The lack of established standards for the use of different staining techniques remains greater attention in the literature on sperm morphological evaluation. There is a need to establish or develop a staining technique that will enable unambiguous and precise analysis of the morphology and morphometry of spermatozoa from different animal species. In addition, a standard should be developed for preparing specimens for morphological evaluation. This would allow for comparison of results between laboratories, which would increase the value of sperm morphology analysis in predicting and evaluating fertility. The aim of the study was to assess the morphometric parameters of boar sperm after using various staining techniques and to verify the hypothesis that the staining technique affects the morphometric parameters of sperm head.

## Material and methods

### Collection of semen samples

Freshly ejaculated semen from 40 insemination boars were used in the study. All boars were in good health and showed normal libido. The ejaculates were collected by the gloved-hand technique.[69] Immediately after collection, the semen was filtered through four layers of sterile gauze into a pre-warmed beaker to remove gel particles. The filtered semen was kept at room temperature until needed for slide preparation. Slides were prepared within 15 minutes of collection. Three ejaculates from each boar were collected at 10-week intervals. From the undiluted semen samples, immediately after collection, smears were prepared on microscopic slides at 37°C. The smears were stained using various staining techniques.

### Staining techniques

Four techniques were used to assess the effect of staining on morphometric parameters of the spermatozoa head: Papanicolaou staining (PAP) and SpermBlue staining (SB), which are recommended for the assessment of human sperm morphology, staining with eosin + gentian complex (EG), which is the most popular staining method for boar sperm morphology and, experimentally, staining with silver nitrate in a colloidal gelatine solution (AgNO_3_). The spermatozoa from fresh, unstained semen were used as a control sample. PAP was performed according to the procedure recommended by the WHO.[14] SB was performed using a commercially-available kit according to the manufacturer’s protocol (Microptic SL, Barcelona, Spain). We used the original SpermBlue Stain, for which the entire procedure takes about 22 minutes. EG was performed according to the procedure described by Kondracki et al. [49]. AgNO_3_ was performed using a modified protocol developed by Andraszek and Smalec [63], based on the basic technique proposed by Howell and Black [58]. From each boar, 300 morphologically normal spermatozoa were evaluated: 60 stained with each technique and 60 unstained (U). In total, 12,000 sperm cells were evaluated.

### Morphometric measurements of sperm head and calculation of sperm head shape indices

The morphometric measurements of sperm head was performed using the MultiScan image analysis system (Computer Scanning Systems, PL) connected with an Olympus BX50 light microscope at 1000 x magnification (100 x oil immersion objective) and Jenoptik ProgRes camera. The system was coupled with a digital camera and the images were stored in computer memory. The analysis was not automatic, as in the case of CASA. Each evaluated sperm was measured manually using measurement software coupled with the MultiScan system.

The area, perimeter, length and width of the sperm head were measured. From these basic morphometric parameters according to Tygerberg criteria, four additional shape indices characterizing the sperm head i.e. ellipticity, elongation, roughness and regularity, were calculated (see Table 1 for formulas). These parameters more precisely characterize the shape of sperm head.

**Table 1:** Morphometric parameters of the sperm head, shape indices and conversion formulas by Maree et al. [4].

**Morphometric parameter**	**Designation**	**Formula**
Length (μm)	L	-
Width (μm)	W	-
Perimeter (μm)	P	-
Area (μm^2^)	A	-
**Shape index**	**Designation**	**Formula**
Ellipticity	E	$\frac{L}{W}$
Elongation	En	$\frac{L-W}{L+W}$
Roughness	Rs	$4\pi *\left(\frac{A}{P^{2}}\right)$
Regularity	R	$\pi *\left(\frac{L*W}{4*A}\right)$

### Statistical analysis

The results were characterized statistically and presented in the form of tables. The effect of the staining techniques on the morphometric parameters and shape indices of the sperm heads was evaluated by one-way analysis of variance using the following mathematical model:

$$Y_{ij}=\mu +a_{i}+e_{ij}$$

Where:

**Y**_***ij***_*−*value of feature
μ –mean for population
**a**_***i***_−effect of i^th^ level of factor (staining technique)
**e**_***ij***_−sampling error

The significance of differences between groups was verified by Tukey’s test at P≤0.05.

Data were analysed by ANOVA using STATISTICA PL 10.0 software (STATISTICA version 10.0, StatSoft Inc., PL)

## Results

### Staining patterns for different staining techniques in boar spermatozoa

**Fig 1:**
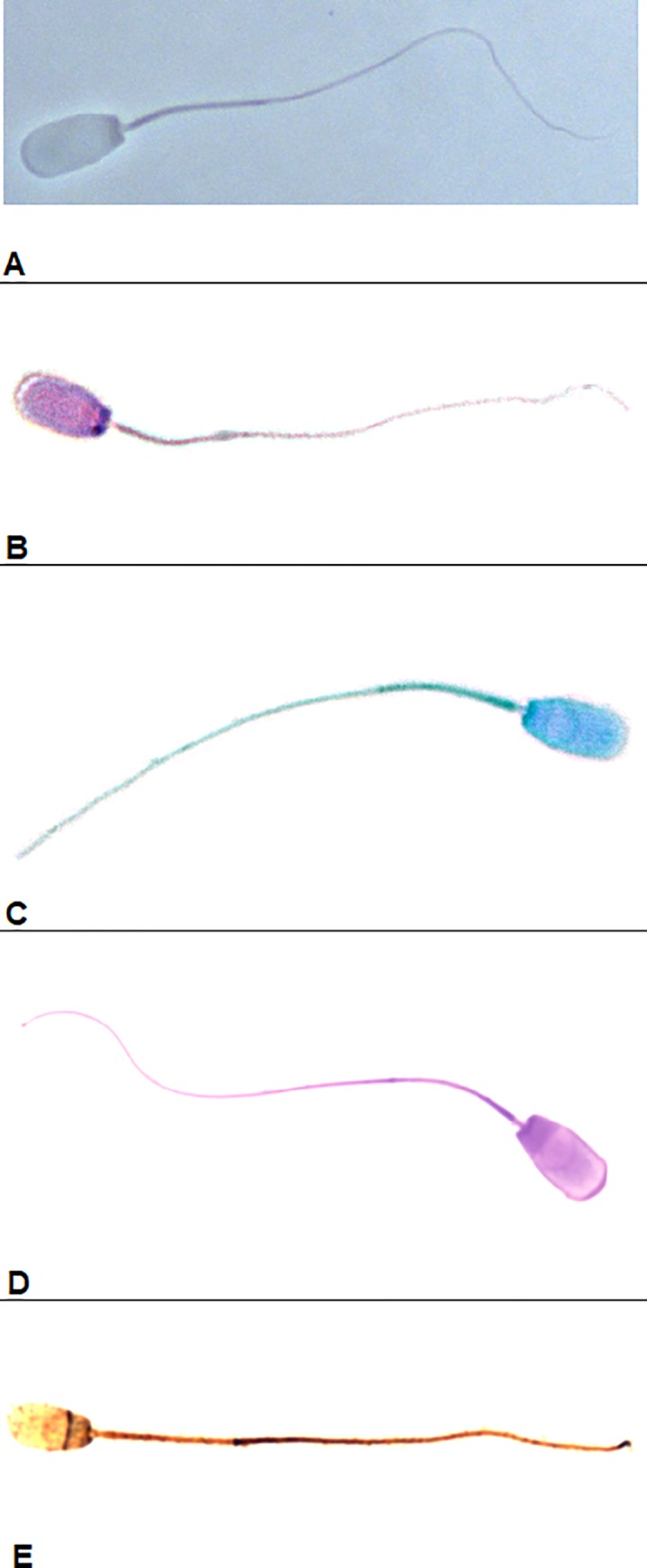
Boar sperm. A–unstained (phase contrast); B–stained with Papanicolau; C–stained with SpermBlue; D–stained with eosin+gentian; E–stained with silver nitrate.

Fig 1A presents spermatozoa from unstained slides in phase-contrast optics (control sample). In PAP (Fig 1B) the heads of the boar spermatozoa were stained light purple. The acrosome was lighter, gradually becoming darker towards the tail, and the extent of the acrosome was difficult to determine precisely. The contour of the head was sufficiently clear, smooth, and easy to identify. The sperm midpiece and tail were pale pink; the end of the tail was difficult to identify and the boundary between the midpiece and tail was imperceptible. The background was light and unstained and did not impede the evaluation.

The SB (Fig 1C)) stains the head of boar spermatozoa blue. The acrosomal part of the head is lighter, but it is not distinct enough for precise measurement of the area and extent of the acrosome. The outline of the head is sufficiently clear to identify and measure. However, the tail becomes a pale greyish blue and it is difficult to distinguish the midpiece from the principle piece of the tail. The background is light and does not impede analysis of the sperm head.

In EG (Fig 1D) the head of the boar spermatozoa are very clearly stained for a pinkish purple colour. The acrosomal part is identifiable. The contour of the head is very distinct and easy to identify. The spermatozoa tail is distinctly stained and identifiable along its entire length, but the boundary of the midpiece is difficult to detect. The background of the slide is light and does not impede analysis.

In AgNO_3_ (Fig 1E), the individual parts of the sperm structure are stained in varying degrees of yellow and brown colours, enabling their precise identification. The sperm head is clearly differentiated into the light (yellow) acrosome part and the dark (brown) distal part. The contour of the head is clearly visible against the background of the slide. Within the tail, the lighter (yellow-brownish) midpiece and the rest of the tail (brownish) are clearly visible. On some slides, where the specimen was too dense, the background was dark with visible grains of silver, but this did not impede accurate measurements.

### Influence of staining techniques on sperm head morphometry

The morphometric values of analysed head parameters for each staining techniques, as well as for unstained sperm heads, are presented in Table 2. Additionally, the differences between mean morphometric values of each staining techniques and unstained spermatozoa were calculated (Table 3). All staining methods have a significant impact on dimensions of the boar sperm head. The morphometric parameters of the sperm heads stained by each technique differ significantly from those of the unstained heads (P≤0.05). The heads stained by PAP had the lowest values of morphometric parameters followed by SB in comparison with unstained heads while sperm heads stained with AgNO_3_ had the highest values followed by EG. The mean values of area, perimeter, length and width of the sperm heads stained by SB were the closest to those of the control group. Contrary, the mean value of area of sperm heads stained by PAP as well as the the sperm head perimeter, length and width stained by AgNO_3_ differed the most. The lowest coefficients of variations (< 4%) were observed for sperm head length in all staining techniques except PAP, as well as in unstained spermatozoa. The highest coefficients of variations (>6%) were noted for head area in unstained spermatozoa and those stained by PAP, SB and EG, and for head perimeter in spermatozoa stained by PAP and AgNO_3_, as well as for head width in all analysed groups.

**Table 2:** Values of morphometric parameters depending on the staining technique.

Morphometric parameters of the sperm head	Statistic	Staining technique				
		PAP	SB	EG	AgNO_3_	U
**Area** **(**μ**m**^**2**^**)**	$\bar{x}$±SD	18.66^a^ ± 1.67	20.25^b^ ± 1.54	27.15^c^ ± 1.80	27.47^d^ ± 1.24	23.65^e^ ± 1.65
	CV%	8.96	7.60	6.64	4.52	9.96
**P (μm)**	$\bar{x}$±SD	20.26^a^ ± 1.29	21.84^b^ ± 1.04	29.02^c^ ± 1.30	30.26^d^ ± 1.83	24.76^e^ ± 1.13
	CV%	6.35	4.78	4.47	6.03	4.55
**L (μm)**	$\bar{x}$±SD	6.59^a^ ± 0.37	7.06^b^ ± 0.27	8.31^c^ ± 0.30	8.82^d^ ± 0.33	7.55^e^ ± 0.28
	CV%	5.59	3.91	3.61	3.82	3.70
**W (μm)**	$\bar{x}$±SD	3.55^a^ ± 0.23	3.74^b^ ± 0.24	4.26^c^ ± 0.27	4.74^d^ ± 0.32	3.84^e^ ± 0.24
	CV%	6.64	6.63	6.36	6.92	6.39

Values with different lowercase letters differ significantly at P≤0.05.

**Table 3:** Differences in means for morphometric parameters between each of the staining techniques and unstained sperm.

Morphometric parameters of the sperm head	Staining technique				
	PAP	SB	EG	AgNO_3_	U
**A (**μ**m**^**2**^**)**	-0.211	-0.144	0.148	0.162	1
**P (μm)**	-0.182	-0.118	0.172	0.222	1
**L (μm)**	-0.127	-0.065	0.101	0.168	1
**W (μm)**	-0.076	-0.026	0.109	0.234	1

In the case of the head area, the lowest coefficient of variation was found for the spermatozoa stained by AgNO_3_ and the highest for PAP stained spermatozoa. The sperm head perimeter, length and width were the least varied in the case of EG. The highest coefficient of variation for sperm head perimeter and length was observed after PAP staining. The most diverse results for head width were found after AgNO_3_ staining.

### Influence of staining techniques on indices of sperm head shape

The head shape indices of unstained boar spermatozoa and spermatozoa stained by different techniques are presented in Table 4. Additionally, the differences between mean values of the indices of each staining techniques and unstained sperm were calculated (Table 5).

**Table 4:** Values of shape indices depending on the staining technique.

Sperm head shape index	Statistic	Staining technique				
		PAP	SB	EG	AgNO_3_	U
E	$\bar{x}$±SD	1.86^a^ ± 0.13	1.89^b^ ± 0.13	1.95^c^ ± 0.13	1.86^a^ ± 0.12	1.97^c^ ± 0.13
	CV%	7.30	6.88	6.78	6.48	6.86
En	$\bar{x}$±SD	0.30^a^ ± 0.03	0.30^a^ ± 0.03	0.32^b^ ± 0.03	0.30^a^ ± 0.02	0.32^b^ ± 0.03
	CV%	10.96	9.88	9.65	9.80	9.20
Rs	$\bar{x}$±SD	0.57^a^ ± 0.04	0.53^b^ ± 0.04	0.40^c^ ± 0.02	0.38^d^ ± 0.04	0.48^e^ ± 0.03
	CV%	7.09	6.90	6.94	11.24	6.90
R	$\bar{x}$±SD	0.98^a^ ± 0.02	1.02^b^ ± 0.03	1.02^b^ ± 0.03	1.19^c^ ± 0.07	0.96^d^ ± 0.05
	CV%	3.00	4.63	3.34	6.24	5.59

Values with different lowercase letters differ significantly at P≤0.05.

**Table 5:** Differences in average values of shape indices depending on the staining technique in comparison with unstained sperm.

Sperm head shape index	Staining technique				
	PAP	SB	EG	AgNO_3_	U
E	-0.056	-0.041	-0.010	-0.056	1
En	-0.063	-0.063	0.000	-0.063	1
Rs	0.188	0.104	-0.167	-0.208	1
R	0.021	0.063	0.063	0.240	1

In comparison with unstained spermatozoa there was no significant difference in head elongation and ellipticity in spermatozoa stained by EG. In other staining techniques, these head shape indices were significantly lower. The lowest ellipticity values were in sperm head stained by PAP and AgNO_3_. In the case of elongation, PAP, SB and AgNO_3_ had the same, lower value. Head roughness was significantly higher in spermatozoa stained by PAP and SB compared to unstained spermatozoa while after EG and AgNO_3_ this head shape index was significantly lower. The lowest value of roughness was observed in spermatozoa stained by AgNO_3_, and the highest in those stained by PAP. Head regularity was significantly higher in all staining techniques when compared with unstained spermatozoa. The sperm heads stained with AgNO_3_ had the highest value for regularity while unstained spermatozoa had the lowest value.

The ellipticity index value closest to that of unstained spermatozoa was found in the sperm stained by EG, while it was the most different in sperm stained by AgNO_3_. The elongation of the sperm head stained by EG was equal to that of the unstained spermatozoa, while the other three staining techniques equally lowered the value by 0.02. The values for head roughness and regularity were closest to that from unstained sample in spermatozoa stained by SB and PAP, respectively, and most different in spermatozoa stained by AgNO_3_. Generally, it can be said that the most shape indices values of sperm head stained by EG were the closest to those of unstained sperm heads, while shape indices values of sperm head stained by AgNO_3_ were the most different.

The low coefficients of variations were observed for head regularity (from 3.00 to 6.24%), and the high for head elongation (from 9.20 to 10.96%) in all four staining techniques and unstained, control sample. The highest coefficient of variation was found for head roughness in spermatozoa stained by AgNO_3_ (11.24%). In the case of head ellipticity, the lowest coefficient of variation was found for the spermatozoa stained by AgNO_3_ and the highest for those stained by PAP. Elongation varied the least in unstained spermatozoa, and the most in spermatozoa stained by PAP. Roughness and regularity were the most varied after use of AgNO_3_. The lowest variation in sperm head roughness value was found after staining by SB and in the unstained, control sample. The lowest variation in regularity was found for the heads of spermatozoa stained by PAP (Table 3).

## Discussion

The significance of the role of sperm morphometry is evidenced by the increasing number of publications describing research carried out all over the world and on various species. For example the relationship between sperm morphometry and fertility in humans has been described by McAlister [34], and Maree et al. [4]. There are studies indicating a relationship between sperm dimensions and fertility in horses [15,43,44,70,71] in which fertility disorders are positively correlated with enlarged sperm heads. The relationship between sperm morphology and fertility has also been studied in boars [10,25,72], bulls [42,73], dogs [17] and foxes [68]. Differences in the size of the sperm heads in fertile and infertile males have been detected in various species, and males whose semen contained sperm with smaller heads have been found to be more fertile.[74] The main sources of variation in sperm morphometry are the sample preparation, fixation method, staining method, microscopic system (optics and camera), and the activity of the technician. All these may affect the repeatability of the analysis, its reproducibility and the comparison of results among laboratories.[74]

It is of great importance that the ideal staining method for sperm morphology assessment should be the one that interferes the least with the structure and size of the spermatozoon, while also clearly showing the boundaries of its head, midpiece and tail.

Our present findings indicate that the choice of staining methods has an impact on the head dimensions of boar sperm. The changes were not uniform for staining techniques used, when the results were compared with unstained spermatozoa from control sample. The spermatoza stained by SB had the closest morphometric parameters of the head to that from the unstained sample, despite the fact that the EG staining is recommended for evaluation of boar sperm morphology in Poland.[49,73,75] The EG staining caused the sperm head to swell, resulting in an increase of all morphometric parameters. However, the stainings which differed the most from the values obtained in unstained spermatozoa were AgNO_3_, which caused the head to swell, and PAP, which caused it to shrink. Similar results were observed in our previous studies on bull and stallion spermatozoa when these methods were used.[42,44] It is important to note that also in these species, the heads of the sperm stained by SB had the closest values of morphometric parameters to those of unstained spermatozoa. These results are in agreement with the pioneering studies of Maree et al. [4] and Van der Horst and Maree [56], which showed the applicability of SB staining in human and different animal species. Each staining technique use a number of different chemical reagents. Each reagent used in staining methods or fixative types can cause either the sperm cell to swell or shrink by penetrating its membrane and influencing the osmotic balance.[4,76–80] Procedures involving higher numbers of stages and chemicals are more likely to damage the sperm cell, resulting in changes in its dimensions.

PAP staining involves the use of over 12 different chemicals, some of which can cause extreme hypoosmotic conditions, and thus shrinking the spermatozoa of different species including humans.[4,42,44,77]

The boar sperm heads stained in our study with AgNO_3_ had the greatest length, width, area and perimeter. The staining procedure is carried out in a gelatine colloidal solution at 60°C, in saturated humidity. It is possible that such conditions may increase the size of spermatozoa due to the hydrophilic properties of the proteins.[63]. As mentioned above, the dimensions of sperm head from different animal species as well as humans stained with SB were the closest to those from fresh, unstained semen. It is probably due to fact that the osmotic potential of the reagents used in this technique is closer to that of the semen and, therefore, the techniques had less effect on the sperm head dimensions than others.[4] So, it seems reasonable to recommend SB staining for the evaluation of sperm morphology and morphometry in pigs, bulls and stallions, because it has the least effect on the basic morphometric parameters of the sperm cell.[42,44,56]

In addition to the basic morphometric parameters of the sperm head, the present study also evaluated four shape indices according to the Tygerberg classification, currently the most precise classification of sperm head parameters.[4,48] It was shown that the sperm stained by EG had the closest shape indices to those of the unstained spermatozoa, while the sperm stained by AgNO_3_ were the furthest. Substantial variation was also observed in the value of individual indices depending on the staining technique. Comparison of the indices characterizing the sperm head for individual staining methods and the control group reveals that unstained sperm heads are more oval and rounded, as indicated by higher ellipticity and elongation values. The sperm heads from the control sample are also more symmetrical, which suggests less interference and less damage to the plasmalemma of the head resulting from the influence of chemical reagents. Considerable differences in head shape indices were also found after using different staining techniques in bulls and stallions spermatozoa.[42,43,45]

There is no doubt that the staining technique should interfere with the cell structure as little as possible while revealing as much detail as possible regarding its morphology. Unfortunately, no such method exists for staining livestock spermatozoa. As mentioned above, SB has the least effect on sperm head dimensions in different species. Unfortunately, it has been shown in a previous study that this technique does not enable a precise determination of the extent of the acrosome in the case of boars. The same was true for stallion and bull spermatozoa. Also PAP staining, the method recommended by WHO for assessment of human sperm morphology, does not work with boars, bulls or stallion spermatozoa. The commonly used EG staining enables assessment of the acrosome only in the sperm of horses [44], while in the case of bulls [43] and boars, as it was presented in this study, the acrosome boundary cannot be unambiguously identified. The use of aniline blue staining to identify sperm structures seems interesting and promising. Although this staining method is used to identify abnormal chromatin condensation, it has been used successfully for detailed morphometric measurements of fox sperm.[68] Unfortunately, in the case of boars and bulls, this staining method does not identify the details of the sperm head.

Among all the staining methods used in the present study, AgNO_3_ seems to be the most promising as for precise identification of the details of the boar spermatozoa structure (the head and its components, midpiece, and tail). This staining has also been successfully used to visualize the sperm structure in spermatozoa of selected farm animals (bull, goat), birds (rooster), insects (drone) and other free-living animals (wild boar, roe deer).[36,42–45,47,63,64,81] Thus, we have shown that AgNO_3_ reveals details specific for a given species and variety, especially with regard to the sperm head. AgNO3 staining highlights the differences in composition of the chromatin of the sperm nucleus: the part containing the acrosome, containing alkaline proteins, stains lighter than the distal part of the head, which contains the remains of acidic proteins and nucleoli [63] Furthermore, this method also clearly shows differences in acrosome integrity (because many details of its structure can be observed), which may occur as a result of spermatozoa damage or ageing.[81] Therefore, in our opinion, although silver staining affects the morphometric parameters of the sperm head more than the other staining techniques used in the study, it can be used for morphological assessment and identification of individual sperm structures.

While certain staining methods are recommended for assessment of human sperm morphology, the search continues for optimal techniques enabling the reliable assessment of animal spermatozoa. Discrepancies in the reaction of spermatozoa to dyes used may result from differences between species or differences between individuals in the resistance of sperm to external factors.[14,74] The structure and arrangement of microfibres of the sperm head may also result in different sperm head dimensions. The cytoskeleton of the sperm head consists of nuclear proteins and the nuclear envelope, which are partially responsible for the formation of the nucleus. Depending on the method of fixation and staining, changes may take place in the arrangement of actin fibres in the sperm head.[82]

Although there are some morphological classifications of sperm defects for some mammalian species.[14] There are no conclusive guidelines recommending specific staining techniques for animal species. One of the systems for sperm morphology classification was developed for cattle.[83–85] This system is also often used to evaluate boar sperm.[50] A slightly different classification of spermaozoa structure has been developed for stallions [86] and the most important sperm defects in poultry have also been defined.[87] Therefore, an important factor to consider when choosing a staining technique is how the staining procedure influences the morphology and dimensions of spermatozoa in comparison to spermatozoa in fresh semen in a given species.[4] Attempts have been made to stain rooster semen [87,88] using the eosin method, which is still recommended for bull semen. However, this method has been found to have a tendency to swell the sperm heads, which disqualifies it for assessment of poultry semen.[41] A very simple technique that has been used for years to evaluate mammalian semen is EG.[49,73] This method stains the sperm head very clearly, but makes it difficult to observe the extent of the acrosome or the midpiece, as demonstrated by the present study and previous studies conducted by our research team.[42,44] Each method has its advantages and disadvantages, and the problem is to select the optimal one for a given species.

Some authors have reported a relationship between sperm morphometry and motility. The shape of the sperm head is an important factor affecting its hydrodynamics, and presumably sperm with more slender and oval heads have greater efficiency of movement. Therefore, we can look for a relationship between the shape of the heads and motility, by observing whether sperm with more oval heads have longer midpieces, whose organelles unquestionably exert an influence on sperm motility.[89] Other studies have demonstrated that males with smaller spermatozoa are more fertile. Ostermeier et al. [90,91] have shown that highly fertile bulls have more elongated but smaller spermatozoa than individuals with lower fertilization capacity.

In conclusion, differences in sperm head dimensions after the application of different staining techniques are due to the fixatives and chemical reagents used in the procedure. These observations lead to the conclusion that it is very important to establish the natural dimensions of the unstained sperm head, and only then to determine the optimal technique and the reference values for this technique. Moreover, it is important to select the right technique for staining the semen of a given animal species, as research by many authors, as well as our own results, indicates that some methods that are useful for one species are not suitable for analysing another. Thus a male may be classified in one laboratory as an individual with normal sperm morphology and in another as having fertility disorders. In the case of boar sperm, the staining technique that least affected sperm head morphometry was SB staining. Nevertheless, although AgNO3 staining affects the morphometric parameters of the sperm head more than the other staining techniques used in the study, it can be used for morphological assessment and identification of individual sperm structures. Although some studies suggest that alternative staining techniques are effective and provide reliable results, others have shown significant differences between staining methods in terms of colour intensity and contrast, but also, most importantly, with regard to the size and shape of sperm. Each of these parameters can have a significant impact on the results of the morphology assessment. These subtle differences in the evaluated specimens are particularly problematic in the assessment of fertility disorders in cases where sperm morphology parameters fluctuate within the limits of reference values.
